# A database with frailty, functional and inertial gait metrics for the research of fall causes in older adults

**DOI:** 10.1038/s41597-023-02428-0

**Published:** 2023-08-25

**Authors:** Sara García-de-Villa, Guillermo García-Villamil Neira, Marta Neira Álvarez, Elisabet Huertas-Hoyas, Luisa Ruiz Ruiz, Antonio J. del-Ama, María Cristina Rodríguez Sánchez, Antonio R. Jiménez

**Affiliations:** 1 Spanish National Research Council, Centre for Automation and Robotics (CSIC-UPM), 28500 Arganda del Rey, Spain; 2 Universidad Rey Juan Carlos, Department of Signal Theory and Communications, Madrid, 28942 Spain; 3 Foundation for Research and Biomedical Innovation of the Infanta Sofía Hospital (HUIS), Department of Geriatrics, Madrid, 28702 Spain; 4 Universidad Rey Juan Carlos, Department of Physical Therapy, Occupational Therapy, Rehabilitation and Physical Medicine, Madrid, 28922 Spain; 5 Universidad de Alcalá, PhD Student, Alcalá de Henares, 28801 Madrid, Spain; 6 Universidad Rey Juan Carlos, Electronic Technology Area, Madrid, 28933 Spain

**Keywords:** Risk factors, Geriatrics, Quality of life, Predictive markers

## Abstract

The GSTRIDE database contains information of the health status assessment of 163 elderly adults. We provide socio-demographic data, functional and frailty variables, and the outcomes from tests commonly performed for the evaluation of elder people. The database contains gait parameters estimated from the measurements of an Inertial Measurement Unit (IMU) placed on the foot of volunteers. These parameters include the total walking distance, the number of strides and multiple spatio-temporal gait parameters, such as stride length, stride time, speed, foot angles and clearance, among others. The main processed database is stored, apart from MS Excel, in CSV format to ensure their usability. The database is complemented with the raw IMU recordings in TXT format, in order to let researchers test other algorithms of gait analysis. We include the Python programming codes as a base to reproduce or modify them. The database stores data to study the frailty-related parameters that distinguish faller and non-faller populations, and analyze the gait-related parameters in the frail subjects, which are essential topics for the elderly.

## Background & Summary

In the current aging population, elderly adults represent the 13.2% of the world’s total population^1^. This population requires specific health and social characteristics^2^. Elderly adults need a clinical emphasis on preserving functional ability and manage functional deterioration since the 4% of this population already presents a significant loss of these capacities^1^.

Declining functional ability is one symptom of frailty, a weakness syndrome associated with aging^3^. Frailty is closely related to the prevalence of falls, which are the second leading cause of unintentional injury death, and death rates are highest in the population over the age of 60 years, whose risk increases with age^4,5^. Around one third of this population have a fall each year and many of them experience recurrent falls^4^.

Fall-risk and frailty assessments are crucial for a multi-factorial intervention to prevent fall-related injuries^6^. Commonly, frailty is diagnosed using the Fried’s criteria^7^, which consists of three questions and two functional tests: (1) Unintentional weight loss in the previous year (more than 5% BMI), (2) Poor endurance and energy, (3) Low physical activity level, (4) Weakness (strength as measured by a hand-held dynamometer) and (5) Slowness (walking speed at 4 meters test). Other frailty scales and functional tests performed in the outpatient clinic include the gait speed, the Timed Up and Go (TUG)^8^ or the Short Physical Performance Battery (SPPB)^9^.

Gait analysis is an alternative method to detect frailty and fall-risk in elderly people, whose use has increased during the last decades^10^. The gold standard for gait analysis is represented by optoelectronic systems that use motion capture techniques, force platforms and electromyography electrodes. Recently, thanks to micro-electro-mechanical systems technology, inertial measurement units (IMUs) have emerged as an option that show a good agreement with the classical gait analysis for some spatio-temporal parameters.

IMUs commonly include a tri-axial accelerometer and gyroscope that measure the linear acceleration and angular velocity. These devices allow to capture kinematic information over extended spaces and time intervals and even outside a laboratory, which makes them optimal to obtain information in real-life conditions. IMUs have already been used for the assessment of fall risk in dynamic tests since falls commonly occur during these motions^11^. The primary gait differences between fallers and non-faller populations are a slower walking speed and shorter step and stride lengths, caused by a fear of falling and decreased muscular strength. However, these temporal differences might be conscious because of the fear, so individuals that do not feel insecure might not present these gait alterations. Other relevant gait parameters to evaluate frailty and fall risks have been analyzed on the basis of *prior* literature^12^.

However, the gait parameters have to be analyzed on studies focused on elderly people in order to assess their significance in the discrimination between faller and non-faller populations and the assessment of their frailty. Public databases are required for the design and optimization of population recognition systems in real-world applications such as the aforementioned gait analysis. To meet this demand for the open database designated for the development of fall-risk and frailty assessment tools among the elder, we detail the GSTRIDE database in this paper.

The data descriptor aims to contribute to the research of frailty and falls causes in relation to gait parameters. The GSTRIDE database includes a large number of participants, that is 163 volunteers, with old age of average 82.6 ± 6.2 years. The volunteers present variability in morphology, functional capacity and frailty evaluation results. The data includes outcomes from common tests for the evaluation of frailty and functional capacity tests, including the Global Deterioration Scale (GDS), the Fried’s phenotype, the Short Falls Efficacy Scale International (FES-I) and the 4-meter walk, TUG and SPPB tests. These evaluation outcomes are combined with the gait parameters obtained from the data recorded with one IMU placed on the foot of volunteers during dynamic walking tests. The results of this analysis include the following metrics: stride time duration, stride length, step speed, percentage of the gait phases (Swing, Stance, Push, Foot-Flat and Load) over the strides, foot angle in the Toe Off and Heel Strike events, cadence, 3D and 2D paths and clearance. We provide these metrics for the steps detected, as well as their average and variance values. The tests outcomes and the gait parameters are included in an MS Excel format file, and in CSV format to ensure usability with any data processing software.

Furthermore, we provide the raw signals recorded with IMUs during these walking tests. The recordings last between 5 and 30 minutes, with an average duration of 21.4 ± 7.1 minutes, depending on the physical resistance of volunteers. The IMU recording files are stored in TXT for ease of use with commonly employed signal computing programs. In this way, this database can be useful to propose new approaches for the study of the gait kinematic parameters estimation from inertial data. We include the Python code for obtaining the gait analysis outcomes from the inertial measurements, which can be edited by software developers.

The socio-demographic data, anatomical, functional and cognitive variables, and the outcome measurements from tests commonly performed for the evaluation of elder people can be connected using the registered data included in the database. By including the results of the health evaluation tests and questionnaires and the inertial and spatio-temporal data, researchers can analyze different regression or classification techniques to identify faller subjects and assess their frailty.

## Methods

### Participants and ethical requirements

One hundred and sixty-three volunteers enrolled in this study: 45 men and 118 women. These volunteers were recruited from the researchers’ family or personal environment, nursing homes, and falls consultation, which they attended after having suffered a fall. Regarding the faller and non-faller populations, 86 volunteers suffered a fall during the last year and the remaining 77 volunteers did not fall during that year. Their age ranges between 70 and 98 years old and they have an average weight of 64.2 ± 13.1 kg and height of 156.8 ± 10.2 cm.

Volunteers presented an average cognitive deterioration status index of 2.05 ± 1.61, according to the GDS and an average frailty index of 8.59 ± 2.73, according to the SPPB test. Their individual specific anthropometric data, age and sex are detailed in the main database file, in the following named “database register”. The file that contains all this information is also named *Database_register* in the Zenodo repository, where the dataset is publicly available for download. The database register also includes the information about volunteers’ falls and their frailty assessment index for the direct connection to the volunteers’ data and measurements.

The study was carried out in the framework of the *Analysis of the Gait Pattern through the Design of an Electronic Prototype and a Monitoring App* project (G-STRIDE, Ref. M2451). The Ethics Committee for Research with Medicines of the Hospital Universitario La Paz approved the study protocol (Ref. HULP: PI-4486, protocol version 1 dated at October 12th 2020, information sheet for the control group and informed consent version 1, dated at December 9th 2020 and information sheet to the participant and informed consent version 2, dated at December 9th 2020). The participation in the study was offered in a voluntary basis. Before the tests, a written informed consent was signed by all participants or their relatives in applicable cases. The protocol provided to all participants details that the information collected from the tests will be entered into a database and the study results will be published for access by the scientific community.

### Frailty and functional assessment

The data include the cognitive, functional and frailty assessment of volunteers. We explain the different tests performed and their evaluation criteria in order of appearance in the database file, named *Database_register*: the GDS questionnaire and the 4-meter walk, frailty assessment, SPPB, TUG, and Short FES-I tests. We provide the outcomes obtained by the volunteers in the different tests and include the evaluation of these outcomes.

The GDS is a validated tool used to screen for primary degenerative dementia signs and the delineation of its stages^13^. The GDS scale consists of a set of progressive 7 questions answered by the volunteers or their caregivers related to their memory and ability deficits. The answers are used to assess the volunteer’s deterioration in the 7 stages of dementia established, from 1 to 7, whose lowest value implies the lowest cognitive deterioration, in which subjects have no subjective complaints of memory deficit. Conversely, the 7^th^ stage indicates the highest deterioration, which indicates the loss of all verbal and basic psychomotor abilities.

The gait speed is a spatio-temporal measurement whose use as a health vital sign is validated^14^. We estimate the gait speed with the 4-meter walk test^15^. To do so, we measure the time spent walking the established distance of 4 meters in a straight line, with the beginning and end of this distance marked on the floor. Volunteers started walking at least one meter behind the initial mark and walked passed the final mark. We set manually the beginning and the end of the test with a chronometer to measure the time required. The speed in this 4-meter walk test is used for the frailty assessment and as one of the tests included in the SPPB, which are explained in the following.

The volunteer’s frailty status is assessed on the basis of the standardized Fried’s phenotype of frailty in elderly adults^7^. This assessment considers five different parameters which are scored as 1, if they are affirmative, or 0, if negative. The final frailty assessment index is the sum of the scores, so it ranges between 0 and 5. The 0 index indicates a robust or non-frail subject, an index of 1 or 2 indicates an intermediate and elevated risk for frailty (prefrail) and an index equal or higher than 3 indicates the volunteer suffers form frailty as the clinical syndrome. Three of these parameters are asked of the volunteers or their caregivers, and they provide their subjective answers. Volunteers are questioned if they noticed an unintentional weight loss during the last year or perceived low energy and resistance. In order to consider the answers affirmative, the weight loss has to be over 4.5 kg and the low energy and resistance has to occur in at least two days per week. They are also asked about their activity level. The answer to this last question is considered affirmative, in the case of men volunteers, if they walk less than three hours per week, whereas in the case of women volunteers, if they walk less than one hour per week.

With regard to the objective measurements, we evaluate the gait speed estimated in the 4-meter walk test and the volunteer’s maximum manual force, measured with a dynamometer. We perform the force tests with an analogical Hydraulic hand dynamometer (Dynamometer JAMAR, Talexco, ES) and a digital dynamometer (Dynamometer MAP 80K1S, KERN&SOHN, GE). Volunteers squeeze the dynamometer with their dominant hand with a rest of several seconds between the two attempts. Some volunteers repeat the tests with the non-dominant hand because the last one is stronger. The measurement reported in the database is the maximum force exerted on the dynamometer regardless of the hand. The gait speed and manual force are considered affirmative if the measurements are lower than the specific thresholds detailed in Table 1, which depend on the volunteer’s sex, height and Body Mass Index (BMI). These reference values can be found in^16^.

**Table 1:** Thresholds established for the frailty evaluation in the gait speed and manual force tests.

Sex	Height [cm]	Speed threshold [m/s^2^]	BMI [kg/m^2^]	Force threshold [kg]
M	≤164	0.50	≤26.4	19.1
	>164	0.43	>26.4	22.9
F	≤152	0.41	≤26.4	11.0
	>152	0.33	>26.4	12.0

The parameters are considered affirmative if the estimated speed and the measured force are lower than the corresponding threshold. The first column indicates the sex of volunteers: M corresponds to male and F to female.

The SPPB is a commonly used protocol for the evaluation of frailty^9^. It includes three tests that evaluate three skills: balance, gait speed, and force, and volunteers score from 0 to 4 points in each test. In this way, the SPPB index ranges from 0 to 12, with the lowest index representing the highest frailty risk and the highest index representing the lowest risk. In the balance test, volunteers score one point if they stand on the floor with the feet together side-by-side for at least 10 s, another point if they stand with the feet in semi-tandem (with the heel of one foot against the side of the big toe of the other one) at least 10 s and another point if they stand with the feet in tandem (feet aligned heel to toe) at least 3.99 s or two points if they stand in tandem over 10 s.

For the gait speed test, we use the speed estimated in the 4-metre test. Volunteers obtain different points according the time required to walk the 4-meter distance: one point if the time spent is longer than 8.70 s, two points if the time is between 6.21 s and 8.70 s, three points if the time is between 4.82 s and 6.20 s and four points if the time is shorter than 4.82 s. If they are unable to perform the test, they score zero points.

We assess the volunteers’ force with the chair stand test. We measure the time spent performing five repetitions of the sit-to-stand movement on a chair, at the maximum possible speed while the volunteers fold their arms across their chest. The scores depend on the time required: if the time is over 60.00 s or they are unable to perform the test, volunteers score zero points; if the time spent is between 16.70 s and 60.00 s, they score one point; if they spend between 13.70 s and 16.69 s, they score two points; if they spend between 11.20 s and 13.69 s, they score three points and if they need less than 11.19 s to carry out the test, they score four points.

Besides the balance test in the SPPB, we evaluate this skill with a dynamic approach by performing the TUG test, which is a different tool to assess balance in elderly people^8^. It consists in measuring the time that takes to get up from the chair without using the arms, walk 3 meters in a straight line, return to the chair and sit down, again with no use of the arms. The time spent is measured manually with a chronometer, starting when volunteers begin their standing up and finishing when they are seated again after walking the trajectory.

Finally, the FES-I test includes questions related to their fear of falling while performing different activities. The questions cover different activities of daily living, such as dressing up, bathing or showering, sitting and standing from chairs, walking through stairs or ramps, taking objects over their heads or from the floor, and going to social events. Volunteers report their indexes between 1 and 4 of their fear of fall, being 1 the value of no fear and 4 the value of very worried about falling. As a consequence, the FES-I test results in an index between 7 and 28 whose lowest value corresponds to the lowest fear.

### Acquisition setup

**Fig. 1:**
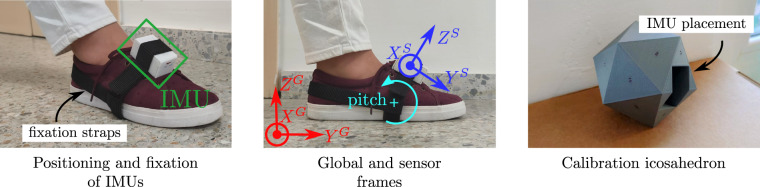
Location of the IMUs on feet and their fixation with elastic straps. The figure on the left depicts the position of the IMUs and their attachment to the foot. The figure in the middle shows the sensor (S) and global (G) reference systems (blue and red, respectively) with the positive direction of the pitch angle relative to the global frame. The figure on the right presents the icosahedron used for the calibration of the IMUs with the gap used to introduce the device.

The GSTRIDE data set includes gait analysis parameters obtained from inertial data. We record these data during a 30-minute walking test with one IMU placed on the top of the volunteers’ foot (see Fig. 1-left). The foot to place the device on was selected by the volunteers according to their preference.

For the walking tests data acquisition, we recorded the inertial data with two kind of devices. On the one hand, we use the IMUs developed *ad-hoc* for this study with an iNEMO inertial module (LSM6DSRX, STMicroelectronics, CH). The development of these IMUs was carried out in the framework of the GSTRIDE project of the Rey Juan Carlos University, with the collaboration of the Infanta Sofía University Hospital and the Spanish National Research Council (URJC/HUIS/CSIC), and they are labeled as CSIC in the database register for simplicity. On the other hand, we recorded the tests with a commercial IMU (Physilog 6 S, GaitUp, CH), labeled as Gaitup in the register. The IMUs measure the angular velocity and linear acceleration, which are stored in one external micro-SD memory and processed *off-line*. The CSIC IMUs record at a sampling rate of 104 Hz with a measurement range of ±2000°/s and ±16 g. The Gaitup IMUs record at a sampling rate of 128 Hz with a range of ±1000°/s and ±8 g. The database includes the specifications from the Datasheet of the sensors used.

Despite the different specifications of the two types of IMUs used, this hardly affects the quality of the estimates. Previous studies indicate that the spatio-temporal estimation results are not very different when different IMUs are used, even with different sampling frequencies^17^. Consequently, there are small differences in estimation accuracy.

Before the walking tests, we calibrate the devices by recording the linear acceleration and angular velocity in a set of static positions on the basis of a previously tested method introduced in^18^. The calibration process requires capturing data from the IMU during an initial 50 second static period and in a sequence of 4–5 second periods at different orientations. To ensure that we obtain the measurements in twenty different and stable positions, we use a icosahendron into which we insert the IMUs for calibration (see Fig. 1-right). Once acquired, these data are processed by the calibration algorithm, which consists of different stages: accelerometer, clock and gyroscope calibration. A more detailed explanation of the calibration can be found at^17^.

### Acquisition protocol

We performed the frailty and functional tests and recorded the motion of volunteers in a continuous session on the day they were available, in the case of the familiar location; on the day the physicians organized the measurement campaign, in the case of the nursing homes; and, in the case of the outpatient clinic, on the days when the patients had an appointment at the hospital, where they were asked if they wanted to participate in the study. For the walking test, we placed the IMU on the instep of the foot of volunteers, who remained standing still. The IMU was placed so that its *Y*^*S*^-axis pointed to the front, the *Z*^*S*^-axis upwards and the *X*^*S*^-axis to the medial or lateral of the body, according to its placement on the left or right foot, respectively, as the axes depicted in blue in Fig. 1. We attached the IMUs to the footwear with elastic straps that wrap around the IMU, encircling the foot at the sole and securing them in the heel area, to ensure that the devices could not move relative to the foot.

The recording protocol started with the switching up of the device, followed by the beginning of the inertial data recording. Volunteers remained still during 30 s and, after this time, they started to walk. The objective was to record the inertial data during 30 minutes, so when volunteers achieved this time of gait, the data recorded was stored and the device was switched off. However, most of volunteers were incapable of walking that long, so their recordings were stopped when they needed to sit and their data were stored in the external memory. At the end of the day, we uploaded the data to a private repository.

Volunteers walked inside or outside buildings, which might affect to the gait motions because of the different surfaces. For that reason, the database register indicates the surface on which the tests were performed. We also take into account the possible influence of carrying out the test on consultation instead of on a familiar environment, so we record this difference in the database register.

### Data processing

We provide the raw inertial data of the gait test together with the calibrated data in TXT format for their direct use in gait analysis. By the IMU calibration, we eliminate the bias of sensors, correct the misalignment and scale the recorded measurements. The data include one calibration file per sensor in TXT format with ten parameters used to perform this calibration.

**Fig. 2:**
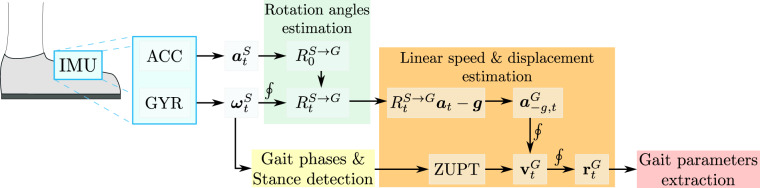
Flowchart of the data analysis performed to obtain the gait parameters. The gait parameters are detailed in Table 3.

Besides the recordings, we provide the result outputs of the gait analysis of the walking test. For the gait analysis, we firstly calculate the rotation matrix from the S to the G frames of reference, ${R}_{t}^{S\to G}$, using the Mahony’s algorithm^19^, as depicted in the overview process flowchart shown in Fig. 2. For the further integration of the linear acceleration, we subtract the gravity force on the accelerometer measurements by the projection of the measured linear acceleration ${{\boldsymbol{a}}}_{t}^{S}$ into the global frame and the elimination of this force in the *Z*^*G*^-axis (see the global and sensor frames in Fig. 1), as ${{\boldsymbol{a}}}_{-g,t}^{G}={R}_{t}^{S\to G}{{\boldsymbol{a}}}_{t}^{S}-{\boldsymbol{g}}$.

Using the gyroscope recordings, we also estimate the time intervals in which volunteers remain still and the different gait phases during the walking time intervals. Gait phase segmentation algorithm is based on algorithms widely used in the literature^?^ and has been validated in previous studies^?^. It consists of identifying still (<0.2 rad/s), movement (>1.41 rad/s) and Foot Flat (<0.5 rad/s) zones by applying thresholds to angular velocity magnitude, while heel-strike and toe-off events are detected as the maximum and minimum pitch values. We exploit the information of the time intervals when the feet barely move, that are when volunteers are still and in the Foot Flat phase of the gait cycle, in the integration of the linear acceleration. To do so, we apply a zero velocity update (ZUPT) approach^20,21^, using a linear interpolation approach between two consecutive stance events to correct the estimated linear speed of the strides. We integrate this corrected linear speed ${{\bf{v}}}_{t}^{G}$ to estimate the linear displacement ${{\bf{r}}}_{t}^{G}$ (trajectory of the foot). Finally, gathering these magnitudes with the time stamps recorded by the IMU, we determine the gait analysis parameters referred to the time and speed of the strides, the different gait phases and the pitch angle during these phases, whose direction is depicted in Fig. 1. Notice the pitch angle refers to the inclination of the IMU in the sagittal plane with respect to the horizontal plane of the global reference system.

The software developed for the IMU data processing to extract the gait parameters is included in the database. The codes are detailed below in the *Code Availability* section.

## Data Records

**Fig. 3:**
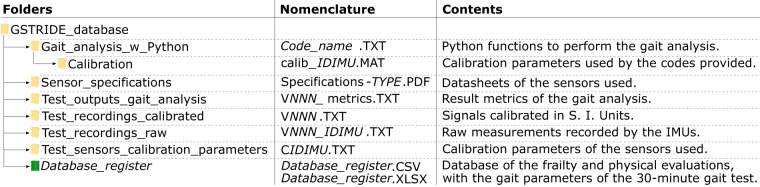
Organization of folders and files in the database. In the nomenclature, *NNN* is the volunteer number, *IDIMU* the IMU identifier, *TYPE* is the kind of sensor, CSIC or Gaitup, and *Code_name* is the name of the codes provided explained in the *Usage Notes* section.

The GSTRIDE full database contains 491 files, which are available at Zenodo^22^, with 10.5281/zenodo.8003441. These files are organized in different folders within the main directory, named “GSTRIDE_database”. The main directory of the database includes the *Database_register* and six folders with different data, as summarized in Fig. 3, which presents the folder organization, their contents and the nomenclature of the files.

The *Database_register* is the database file that contains the outcomes of the frailty and functional evaluation of volunteers and the gait parameters of the 30-minute walk test. This file is stored as CSV, to ensure its use with any data processing software, and as an MS Excel (XSLX) table with colored header and comments for an easy understanding of the data. We provide a fully explanation of this formatted file in the *Database register* section.

Regarding the folder organization, the four folders named as “Test_*”, followed by different details, include the raw and calibrated data, the parameters used to calibrate the raw signals and the outputs of the gait analysis. The remaining two more folders, named “Sensor_specifications” and “Gait_analysis_w_Python”, contain the datasheets of the sensors and the codes provided for gait analysis, respectively. All data files in these folders are stored as TXT, to ensure their use with any data processing software, and as PDF.

### Database register

**Fig. 4:**
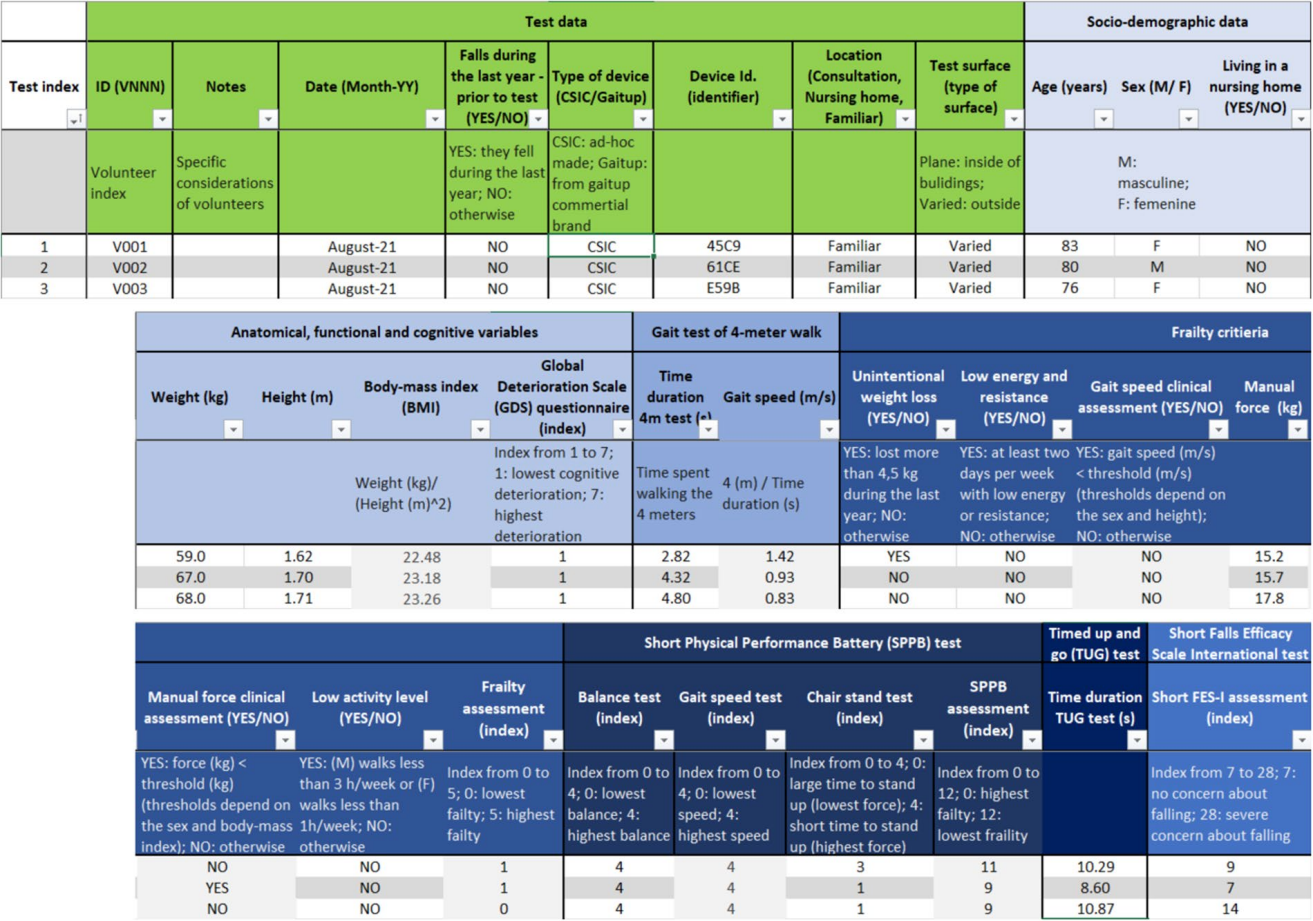
Sections of the tests, socio-demographic and anatomical data, and outcomes of the evaluation tests performed. The first and second rows are the name of the sections an of the parameters, respectively. The third row contains a brief explanation of these parameters, which are detailed in this work.

**Fig. 5:**
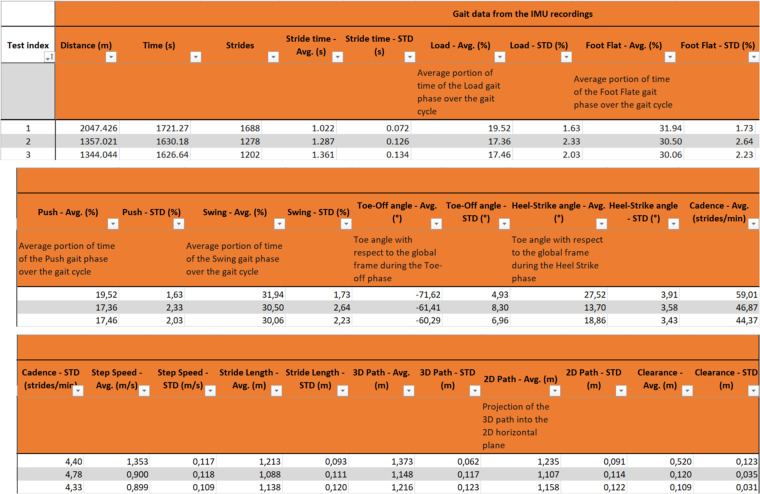
Gait parameters from the 30-minute walking test. The first and second rows are the name of the section and of the gait parameters, respectively. The third row contains a brief explanation of the parameters, detailed only in the average (Avg) fields because they are similar for their standard deviation (STD).

This work contributes to the study of the functional and frailty assessment of elderly people and its relationship with their gait parameters obtained from inertial measurements, which are provided in the *Database_register* file. This file includes the data of volunteers in rows for each test performed, whose outcomes are separated into different sections by columns. The first row indicates the name of the sections, the second one specifies the corresponding parameters and the third row explains these parameters, as Figs. 4, 5 show. The following rows are outcomes of the tests and the gait analysis parameters.

With respect to the organization of sections, the first columns are referred to the test details, presented in the “Test data” section, the socio-demographic data, which are in the “Socio-demographic data” section, and the anthropometric and cognitive parameters, included in the “Anatomical, functional and cognitive variables” section (see Fig. 4). These data are followed by the results of the frailty and functional evaluations, located at the sections named as the corresponding test, as depicted in Fig. 4.

The first column in the *Database_register* corresponds to the test index. This index is related to the volunteer number, included in the “Test data” section. The following columns of this section include the specific consideration of the volunteers and tests, the date when the tests were performed and the information about the possible falls during the previous year, in relation with the walking test, the type of device used and its identifier, the location where the test was performed and the type of surface on which the volunteer walked. The tests were performed in three locations involving different ambulation difficulties. These locations are labeled in the database as “Nursing home”, which indicates that patients walked on known environments, “Consultation”, meaning that patients walked in the hospital but not in the streets, or “Familiar”, for the cases that patients walked in the surroundings of their houses.

The “Socio-demographic data” section includes the parameters of age and sex and the information about whether the volunteers live in a nursing home. The next section, named “Anatomical, functional and cognitive variables”, details the weight, height, body-mass index and GDS index of the volunteers.

The following sections are named as the corresponding assessment test performed, whose evaluation criteria are detailed above in the *Frailty and functional assessment* section. In this way, the “Gait test of 4-meter walk” section contains the time spent to walk the 4 meters and the gait speed. The “Frailty criteria” section includes the parameters of the Fried’s phenotype^7^: the unintentional weight loss, the low energy and resistance, the gait speed, the manual force outcome and evaluation, the low physical activity level and the global assessment of the volunteers’ frailty according to these parameters. The “Short Physical Performance Battery (SPPB) test” section presents the scores obtained by the volunteers in the three tests of balance, gait speed and chair stand, and their final assessment. The “Timed up and go (TUG) test” section provides the time spent in the performance of the TUG test. Finally, the last section of these evaluations correspond to the outcomes of the FES-I test.

Conversely, the second half of the columns contain the gait parameters obtained from the inertial data recorded in the 30-minute walking test. Figure 5 shows the organization of this section, named “Gait data from the IMU recordings”. The three first gait parameters correspond to the distance walked during the walking test, the time the volunteers were walking, as some of them were not robust enough to walk the established time, and the number of steps taken in the test. The remainder are the average and standard deviation of the estimated gait cycle (stride) parameters. These parameters are detailed in the *Processed data* section as they are statistical metrics of the results provided for each volunteer in the “Test_outputs_gait_analysis” folder.

### Raw data

The raw data consist in 158 files of the angular velocity and linear acceleration measured during the walking tests. These data include 11 recordings of a duration between 5 minutes and 10 minutes, 26 recordings between 10 minutes and 15 minutes, 28 recordings between 15 minutes and 20 minutes, 41 recordings between 20 minutes and 25 minutes, 39 recordings between 25 minutes and 30 minutes and 13 recordings longer than 30 minutes. The information in these files is labeled as “accA[a.u.]” and “gyrA[a.u.]”, where “A” refers to the corresponding measurement axis and “[a.u.]” to arbitrary units. The information given with each label is detailed in Table 2.

**Table 2:** Label of the columns in the TXT files of the raw data and the calibration parameters with their units and description, where D.N. = Dimensionless Number.

	Column label in the files	Unit	Description
Raw data	accA	a.u.	Linear acceleration with the influence of the gravity force measured with the accelerometer before the calibration. “A” indicates the corresponding coordinate: X, Y and Z, which correspond to the lateral, frontal and vertical directions.
	gyrA	a.u.	Angular velocity measured by the gyroscope before the calibration.“A” indicates the corresponding coordinate: X, Y and Z, which correspond to the lateral, frontal and vertical directions.
Calibration parameters	SSR_biasVector	a.u.	Bias vector of the sensor indicated in the SSR field, which is the accelerometer (ACC) or the gyroscope (GYR).
	SSR_misalignmentM_colN	D.N.	Misalignment matrix of the sensor indicated in the SSR field, which is the accelerometer (ACC) or the gyroscope (GYR). The matrix is divided in the three columns ordered as the “N” number.
	SSR_scalingM_colN	D.N.	Scaling matrix of the sensor indicated in the SSR field, which is the accelerometer (ACC) or the gyroscope (GYR). The matrix is divided in the three columns ordered as the “N” number.
	clockError	D.N.	Scaling factor to adjust the time stamp to the actual sampling rate of the sensors. The column contains this scaling factor followed by two zeros.

The raw data are located in the folder named “Test_recordings_raw”. These files are called with the nomenclature V*NNN_IDIMU*, where *V* refers to “volunteer”, *NNN* is the number of identification of the volunteer, from 1 to 163 and *IDIMU* is the identifier of the IMU used to record the test. As an example, *V001_45C9.TXT* contains the recording of volunteer 1, which was recorded by the IMU 45*C*9. The *IDIMU* is similar to the name of the test sensor calibration parameters in the “Test_sensors_calibration_parameters” folder, but preceded by a “C” letter, as shown in Fig. 3. Table 2 details these calibration parameters and their organization related to the column labels in the files.

### Processed data

The processed data consist in the calibrated recordings and the gait analysis results. The calibrated recordings are in the folder named “Test_recordings_calibrated” and follow the nomenclature V*NNN* (see Fig. 3). These data are the measured angular velocity and linear acceleration signals already in the International System of Units, in radians per seconds and meters per squared seconds, respectively, so their columns are labeled as “accA[m/s^2^]” and “gyrA[rad/s]”. Table 3 includes the explanation of these data. Notice we eliminate the bias and correct the misalignment of sensors *prior* this conversion of units.

**Table 3:** Label of the columns in the TXT files of the calibrated data, with their units and description.

Column label in the files	Unit	Description
accA	m/s^2^	Calibrated linear acceleration with the influence of the gravity force. “A” indicates the corresponding coordinate: X, Y and Z, which correspond to the lateral, frontal and vertical directions.
gyrA	rad/s	Calibrated angular velocity. “A” indicates the corresponding coordinate: X, Y and Z, which correspond to the lateral, frontal and vertical directions.

With respect to the gait analysis results, the files in the folder named “Test_outputs_gait_analysis” contains the output metrics for the steps detected in the test performed by each volunteer. These files follow the nomenclature V*NNN*_metrics, as in the raw and calibrated data (see Fig. 3).

**Fig. 6:**
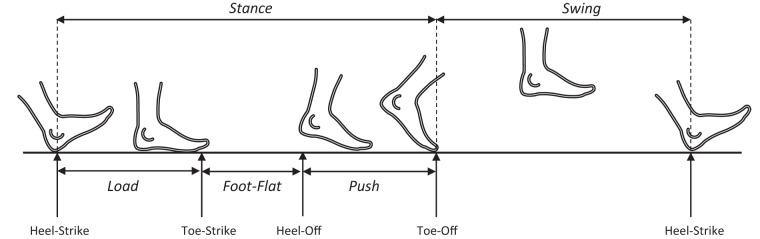
Temporal events and gait phases and sub-phases during a stride. Figure from Fig. 2(a) in^28^.

The metrics provided for each step consist in the time duration of the gait cycles, the percentage of cycle in the different phases (Load, Foot-Flat, Push and Swing), the pitch angle of the foot in the Toe Off and Heel Strike motions, the cadence, the gait speed, the stride length, the lengths of the 3D and 2D trajectories, and the clearance. Figure 6 shows a schematic of the different gait phases and the events that delimit them. Table 4 describes the gait analysis parameters in relation with the labels in the files and their units.

**Table 4:** Label of the columns in the TXT files of the gait analysis outputs.

Column label in the files	Unit	Description
Num. Step	s	Number of the *n* step identified in the experiment, with $n=0\ldots N-1$ where *N* is the number of steps detected.
Stride time	s	Time spent to walk the stride *n*. It is calculated by the time elapsed between the beginning and the end events of a stride. $ST=Time(end)-Time(ini)$
Swing	% gait cycle	Time percentage of the stride *n* during which the foot is in the Swing phase, see Fig. 6. It is calculated as the elapsed time between Toe-Off and Heel-Strike events. $Swing=[Time(TO)-Time(HS)]\times 100\div\,ST$
Load	% gait cycle	Time Percentage of the stride *n* during which the foot is in the Load phase, see Fig. 6. It is calculated as the elapsed time between the Heel-Strike event and Toe-Strike (TS). $Load=[Time(TS)-Time(HS)]\times 100\div\,ST$
Foot Flat	% gait cycle	Percentage of the stride *n* during which the foot is in the Foot Flat phase, i.e. the foot is completely in contact with the ground, corresponds with central part of the stance phase, see Fig. 6. It is calculated as the elapsed time between Toe-Strike (TS) and Heel-Off (HO) events detected. $Foot-Flat=[Time(HO)-Time(TS)]\times 100\div\,ST$
Push	% gait cycle	Percentage of the stride *n* during which the foot is in the Push phase, see Fig. 6. It is calculated as the time elapsed from the Heel-Off (HO) until the Toe-Off occurs. $Push=[Time(TO)-Time(HO)]x100\div\,ST$
Toe Off angle	°	Pitch angle of the foot in the Toe Off moment of the stride *n*. $TOAngle=Pitch(TO)$
Heel Strike angle	°	Pitch angle of the foot in the Toe Off moment of the stride *n*. $HSAngle=Pitch(HS)$
Cadence	strides/minute	Number of strides per minute according to the time spent in walking the stride *n*. $Cadence=60\div\,StrideTime$
Speed	m/s	Average linear speed during the stride *n*. $Speed=StrideLength\div\,StrideTime.$
Stride Length	m	Length between the initial and end points of the stride *n*. It is calculated as the increment in the XY position (horizontal plane) between the initial and final points of the stride. $SL=\sqrt{\left({\left[X(end)-X(ini)\right]}^{2}+{\left[Y(end)-Y(ini)\right]}^{2}\right.}$
3D Length Path	m	Length of the 3D trajectory of the stride *n*. It is calculated as the cumulative three-dimensional displacement (XYZ) made during the stride.
2D Length Path	m	Length of the projection of the 3D trajectory of the stride *n* into the horizontal plane. It is calculated as the cumulative horizontal displacement (XY) made during the stride.
Clearance	m	Elevation of the foot during the swing phase of the stride *n*. It is calculated as the maximum height that the foot reaches during swing phase. $Clearance=max\left(Y(ini:end)\right)$

Their units are indicated and the parameters are described in terms of their definition and the calculation performed to obtain them.

On the basis of the parameters obtained for each volunteer, we provide the average and standard deviation of the individual result outputs. To obtain these metrics, we eliminate the strides longer than 1.4 times the median length or lower than 0.36 times this median.

## Technical Validation

### Sensor placement

Volunteers wore closed footwear in order to avoid discomfort on the foot that might affect their normal gait pattern. The IMUs were placed on the top of the foot, maintaining always the orientation of the *Y*^*S*^-axis pointing to the frontal direction and the *Z*^*S*^-axis to the upwards direction (see Fig. 1), as previously mentioned. The researchers or specialized physicians placed the sensors to ensure their placement in the same location and orientation.

### Missing data

The register includes data of volunteers that were unable to perform all the frailty and functional evaluation tests, so these are indicated with the label “incapable” in the corresponding test. A total amount of 45 parameters are labeled in this manner. Conversely, cells that include only a dash, “-”, indicate a lack of these data. Only 5 parameters are missed: 2/163 results of the manual force, 1/163 measurement of the time in the TUG test and 2/163 outcomes of the short FES-I questionnaire.

Also, due to problems in the storage, the raw and calibrated data of five volunteers (V142, V143, V157, V158 and V159) are loss in the database. However, the output results of their gait analysis and their metrics are included in the database register since they were uploaded in an external repository before the problem occurred. We include these patients in the database register, since the data referred to the functional and gait tests are health data on these patients are available and of interest for research on falls and frailty in the elderly.

### Comparison with published data sets

This database focuses on the fall-risk and frailty assessment of elderly people. This is a relevant topic of growing interest because of its relevance on the fall prevention, one of the most incident accidents among the elderly population^23^.

The age of the participants is relevant since this data set only includes elderly adults, which is the main objective population of this research. Subject number is an important technical for significant metrics and we provide enough data for the development of statistical models and the application of Machine Learning algorithms. Compared to other databases that include inertial data of gait, such as^24–27^, GSTRIDE outnumbers the participants. The data set includes 3,481.6 minutes (58.0 hours) of recordings of inertial data and most participants have a relatively large amount of data collected, which implies a higher amount of data than other studies focused on the older adults^27^. Furthermore, volunteers performed the walking tests in out-of-the-lab environments, that is a worth mentioning difference with other databases that include IMU recordings for the study of the frailty and fall risk^27^.

## Usage Notes

The research lines this work contributes into, the fall-risk assessment and frailty evaluation, are essential for the development of prevention tools focused on the elderly people. Conventionally, the health status evaluation of this population is performed by questionnaires or tests in consultation. However, the answers to the questionnaires are commonly subjective and the white coat effect is widely known to influence into the measurements when they are taken by physicians. This database includes data for the development of alternatives focused on the gait analysis with IMUs, whose measurements can be obtained during the common daily routines of volunteers.

**Fig. 7:**
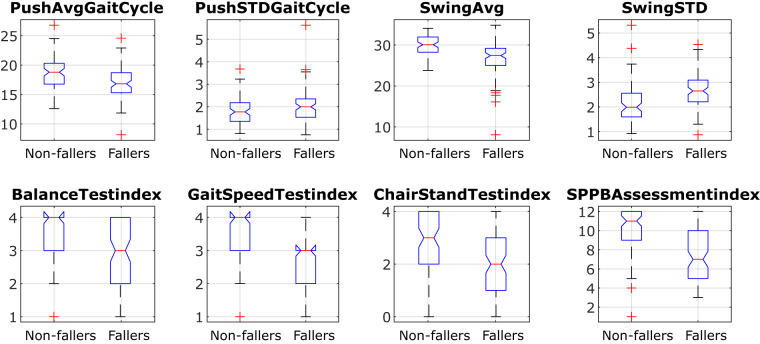
Boxplots of the average and standard deviation of the time spent in the Push and Swing gait phases and the SPPB scored points. The names correspond to the name of the columns in the CSV and XSLX files, explained in Table 4. The boxplots presents the features divided into the faller and non-faller populations. The notches indicate the intervals that, if separated, define the 5% significance level differences of the medians.

As an example, Fig. 7 shows the *boxplots* of different features in the Database for the faller and non-faller populations. The features presented are the average and standard deviation of two gait parameters (the percentage of time of the stride of the Push and Swing phases) and the outcomes of the SPPB assessment. These boxplots show how the percentage of time in the Push phase (labeled as Toe Off because of its name in the Database register file) is a gait parameter that differentiates to a lesser extent the faller subjects than the Swing time percentage. It can also be seen how the Gait Speed and the final SPPB Assessment index discern to a greater extent both populations than the balance or chair lift test, which corresponds to the strength assessment.

In addition, we provide the software developed to analyze the walking data and obtain the relevant gait parameters provided in the register. The project was developed using *Python 3.10.4*. The main code is named *main.py* and it allows the definition of the volunteers to analyze and the possible outputs and the data visualization. We explain the function of each code in order to give developers an overview of the process for them to change our approach if needed. This main code manages the following codes and functions:

- *readLogFileArduinoCalib.py* imports the data and perform the calibration of the recording signals,
- *MahonyAHRS.py* contains the implementation of Mahony’s AHRS algorithm to estimate the rotation matrix that we use to obtain the Euler angles and the quaternions,
- *StepDetection_MultiCorrector_Pitch.py* identifies the relevant gait events, being specially relevant the Foot Flat phase and the still time intervals,
- *INS_ZUPT.py* estimates the speed and linear displacement by exploiting the intervals when volunteers remain still and the Foot Flat phase of strides to correct the estimations,
- *Gait_Parameters_Estimation.py* calculates the gait analysis parameters from the estimated gait events, the corrected speed and linear displacements and the pitch angle.

**Fig. 8:**
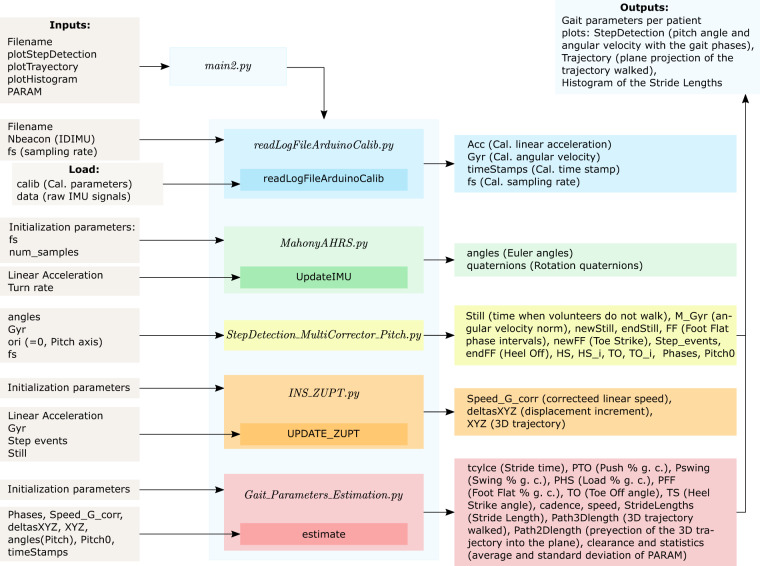
Inputs and outputs of the codes included in the database. The text between brackets describe the relevant parameters or relate them with the name of the parameter in this data descriptor. PARAM is the input variable that allows the selection of the parameters to study. The abbreviation Cal. indicates calibration and g. c. means gait cycle.

The inputs and outputs used by the codes and functions are schematized in Fig. 8. Note that these codes use the raw data from IMUs and not the already calibrated signals since the first step is to eliminate the bias, correct the misalignment and escalate the measurements of angular velocity and linear acceleration and to convert them into rad/s and m/s^2^, respectively. For this calibration, the *readLogFileArduinoCalib.py* code uses the MAT files in the “Gait_analysis_w_Python\ Calibration” folder. We provide twice these data because the codes uses the MAT files but not every software can use this format, so we include them also as TXT files.

**Fig. 9:**
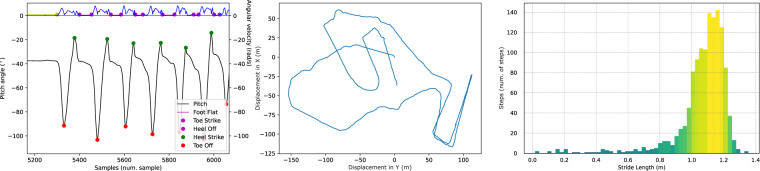
Figures resulting for the volunteer V002 by running the codes provided in this data set. The picture on the left depicts the signals of norm of the angular velocity (M_Gyr), the pitch angle and the gait events of Heel Strike and Toe Off and Foot Flat phase detected. The figure shows how Foot Flat interval correspond to those time intervals when the angular velocity is close to 0 rad/s. Yellow circles with a yellow line between them indicate the beginning and end of still periods. The picture on the middle depicts the estimated 2D path of the complete walking test. Finally, the picture of the right shows the histogram of the number of strides per stride length.

To demonstrate an example use of our database with the codes provided, Fig. 9 shows the output plots obtained with the recordings of one volunteer. According to the gait events and phases detected, the pitch angle calculated and the 2D trajectory estimated (see Fig. 9-left and-middle), we obtain the parameters described in Table 4 in an external CSV file. Figure 9-left includes yellow dots to point out the beginning and the end of the Still periods, during during which people are standing without walking. Time intervals during which people do not walk are not taken into account in the estimation of gait parameters.

Figure 9-right allows the study of the distribution of strides of volunteers. We established the thresholds for the gait analysis provided in the database register to eliminate those steps that are outside the normality range displayed. In this way, we eliminate the strides that do not respond to the normal gait pattern.

## Data Availability

We provide the gait analysis software used to obtain the parameters provided in the register. The Python codes, which are detailed in the Usage Notes section, can be found in the folder named “Gait_analysis_w_Python”. To generate the calibrated signals from the IMU raw measurements, we use a calibration function similar to the one provided in the *readLogFileArduinoCalib* code. For a correct functioning, the distribution of folders must remain as organized in Zenodo^22^ or the file paths have to be updated to their new locations.
